# Circular RNA circ_0111277 attenuates human trophoblast cell invasion and migration by regulating miR-494/HTRA1/Notch-1 signal pathway in pre-eclampsia

**DOI:** 10.1038/s41419-020-2679-6

**Published:** 2020-06-25

**Authors:** Yuhua Ou, Liqiong Zhu, Xiangcai Wei, Shiyu Bai, Manqi Chen, Hui Chen, Jianping Zhang

**Affiliations:** 1 Department of Obstetrics and Gynecology, Guangdong Women and Children Hospital, Guangzhou 511400 Guangdong, China; 2 Department of Obstetrics and Gynecology, Sun Yat-Sen Memorial Hospital, Sun Yat-Sen University, Guangzhou 510120 Guangdong, China

**Keywords:** Mechanisms of disease, Diseases, Pathogenesis

## Abstract

Mounting evidence has revealed that impaired spiral artery remodeling, placental dysfunction, and inadequate trophoblast invasion are closely correlated with the etiology and pathogenesis of pre-eclampsia (PE). Moreover, defective trophoblast invasion may trigger poor maternal–fetal circulation and placental hypoxia, leading to PE. However, the detailed molecular pathology of PE remains unclear. Although circRNAs, as a new type of stable and abundant endogenous noncoding RNA, have been proven to be essential to the pathogenesis of various diseases, their role in PE requires further verification. In this context, it is necessary to unveil the roles of circRNAs in regulating the migration and invasion of extravillous trophoblasts. In this study, using quantitative real-time PCR, we confirmed that hsa_circ_0111277 was upregulated in PE placentas relative to the level in normal pregnancy placentas. In addition, positive correlations between hsa_circ_0111277 expression and PE-related factors (proteinuria level at 24 h and placental weight) were identified by Pearson’s analysis based on the clinical data of 25 PE patients. Moreover, fluorescence in situ hybridization analysis illustrated that circ_0111277 was preferentially localized within the cytoplasm. Mechanistically, circ_0111277 sponged hsa-miR-494-3p in trophoblast cells to attenuate the latter’s repression by regulating HTRA1/Notch-1 expression. In conclusion, trophoblast cell migration and invasion were shown to be promoted and modulated by the hsa_circ_0111277/miR-494-3p/HTRA1/Notch-1 axis, which provides useful insight for exploring a new therapeutic approach for PE.

## Introduction

Pre-eclampsia (PE) is a hypertensive disorder of human pregnancy, which manifests as new-onset hypertension and proteinuria after 20 weeks of gestation^1^. This gestational hypertension disease occurs in 3–8% of pregnant women, leading to high fetal morbidity and pregnancy-induced mortality^2,3^. Unfortunately, the etiology and pathogenesis of PE are still far from clear. However, increasing evidence has confirmed that impaired spiral artery remodeling, placental dysfunction, and inadequate trophoblast invasion may play pivotal roles in the occurrence and development of PE^4–6^. In addition, either extensive or shallow trophoblastic invasion of extravillous trophoblasts (EVTs) at the maternal–fetal interface has been recognized as a major trigger of placentation failure, ultimately contributing to the occurrence of PE^7,8^. Moreover, it has been confirmed that restricted EVT cell migratory activity in maternal decidua may impede trophoblast function, thus initiating PE^9^. Consequently, it is essential to unveil the pathophysiological mechanisms that underlie dysregulated migration and invasion of EVTs.

Endogenous noncoding RNA comprises more than 90% of the transcriptome in humans, and circular RNA (circRNA) is a typical subclass of it. CircRNAs are produced by precursor mRNA backsplicing and present a covalently closed loop structure that lacks free 3′ and 5′ ends^10,11^. Such circular transcripts were long considered to be merely aberrant splicing byproducts. However, circRNAs are now generating more attention due to their essential roles in various physiological and pathophysiological processes, including sponging microRNA (miRNA), modulating parental gene expression, and regulating alternative splicing^12–14^. Many studies have suggested that noncoding RNA participates in the pathogenesis of PE^15,16^. A recent study by Hu et al.^1^ suggested that hsa_circ_0036877 function as a ceRNA and serve as a potential novel blood biomarker for early PE. Currently, the role of circRNAs in PE has been rarely studied, and the pathology of it remains uncovered, so further studies of this issue are needed.

Pregnancy-associated plasma protein A2 (PAPPA2), which was identified as a homolog of PAPPA in the metzincin superfamily of pappalysins, has attracted substantial attention due to its involvement in the pathogenesis of PE^17,18^. It has been reported that PAPPA2 is upregulated in severe early onset PE^19^. Besides, Dimitriadis et al. confirmed that the impairment of EVT invasion, spiral artery remodeling, and placentation derived from IL-11 can be successfully reversed by silencing PAPPA2^17^. However, there has still been inadequate research aimed at its underlying mechanism, so further confirmation of this is still required. Here, to further exploit the roles of circRNAs together with PAPPA2 in the pathogenesis of PE, circPAPPA2 (hsa_circ_0111277) was applied in this study to determine the molecular mechanisms controlling trophoblastic invasion and migration in PE. Our results showed that hsa_circ_0111277 was significantly upregulated in PE placentas. In addition, Pearson’s correlation analyzes confirmed that hsa_circ_0111277 was positively correlated with both proteinuria level and placental weight. Knockdown of hsa_circ_0111277 expression can efficiently promote trophoblastic invasion and migration, providing a novel strategy for treating PE. Mechanistically, hsa_circ_0111277 was proven to bind to and sponge miR-494, thus regulating the expression of the miR-494 target gene HTRA1 along with its downstream signaling pathway. In this study, hsa_circ_0111277 was knocked down by siRNA to relieve the suppression of miR-494, thereby promoting EVT invasion and migration, ultimately modulating PE progression. Collectively, our data confirmed that hsa_circ_0111277 exerts inhibitory effects on trophoblast invasion and migration through the miR-494-3p/HTRA1/Notch-1 axis, which should deepen our understanding of the mechanism underlying PE and facilitate the search for a novel therapeutic regimen for PE.

## Results

### hsa_circ_0111277 expression was upregulated in PE placentas

**Fig. 1:**
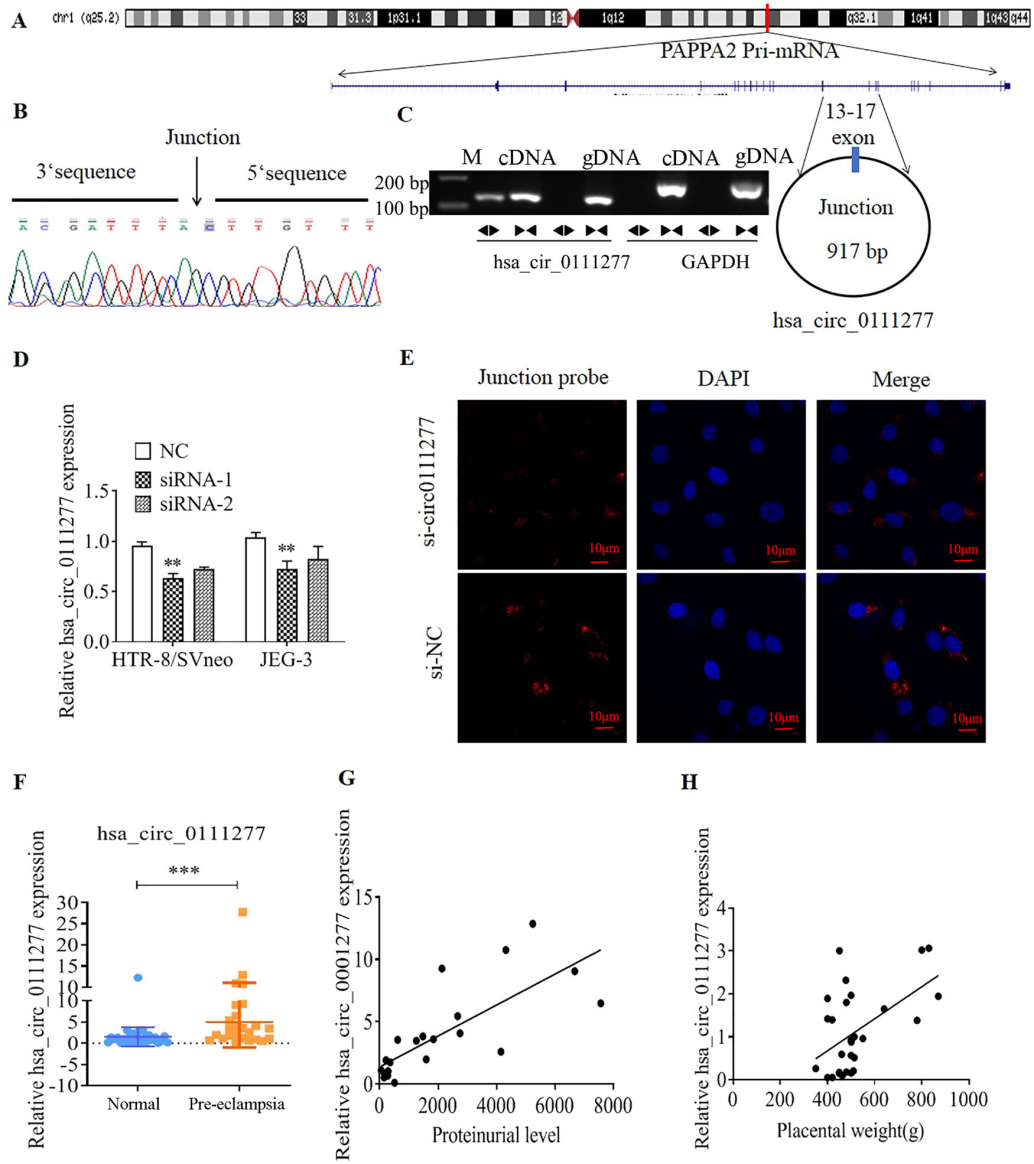
Characterization of circ_0111277 and its correlation with PE. **a** A schematic illustration of the genomic location and splicing pattern of hsa_circ_0111277. **b** The sequence of the splice junction as confirmed by Sanger sequencing. **c** The existence of hsa_circ_0111277 was confirmed by gel electrophoresis. circ_0111277 was amplified by divergent primers in cDNA, but not gDNA. GAPDH served as a linear control. **d** hsa_circ_0111277 in HTR-8/SVneo and JEG-3 cells transfected with siRNA-1 or siRNA-2 was detected by qPCR analysis, respectively. **e** The subcellular location of hsa_circ_0111277 in JEG3 cells transfected with si-circ_0111277 was examined by fluorescence in situ hybridization. **f** hsa_circ_0111277 levels in PE placentas compared with those in normal pregnancy placentas were determined by qRT-PCR analysis. ***P < 0.05 vs. normal pregnancy placentas. Relationships of hsa_circ_0111277 expression with proteinuria level at 24 h (**g**) and placental weight (**h**) in 25 PE patients were subjected to Pearson’s analysis.

To obtain insights into the role of hsa_circ_0111277 in the etiology and pathology of PE, hsa_circ_0111277 was generated by circularizing products in the range of exons 13–17 of the mRNA NM_020318.3 (Fig. 1a). As displayed in Fig. 1a, hsa_circ_0111277 is 917 base pairs (bp) in length, which is derived from five exons located in its host gene PAPPA2. PAPPA2, a noncovalently linked dimer of two 220-kDa subunits, was previously identified to be dysfunctional in PE placenta^20^. The sequence of the splice junction was next confirmed by Sanger sequencing, which was in line with CircBase data (Fig. 1b). The presence of hsa_circ_0111277 in placental tissues was then confirmed by performing polymerase chain reaction (PCR) analysis and agarose gel electrophoresis (Fig. 1c). As depicted in Fig. 1c, the result showed that hsa-circ-0111277 can only be amplified in cDNA by adopting divergent primers as compared with genomic DNA (gDNA). The two specific siRNAs targeting hsa-circ-0111277, namely, siRNA-1 and siRNA-2, were separately applied to knockdown hsa-circ-0111277 in HTR-8/SVneo and JEG-3 cells, and qPCR analysis showed that hsa-circ-0111277 levels were downregulated to a greater extent by siRNA-1 than by siRNA-2 (Fig. 1d). Thus, siRNA-1 (referred to as si-circ-0111277) was utilized in subsequent research to achieve higher transfection efficiency. As demonstrated in Fig. 1e, fluorescence in situ hybridization (FISH) analysis for hsa_circ_0111277 was carried out in JEG3 cells under different treatments. The results revealed that circ-0111277 was preferentially localized within the cytoplasm in JEG3 cells treated with nonsense siRNA (si-NC). Meanwhile, the red fluorescence intensity of junction probes of hsa-circ-011127 that represented the subcellular location of hsa_circ_0111277 was dramatically alleviated in JEG3 cells upon treatment with the vector of si-circ_0111277, implying that such a vector specifically targeted circ_0111277. To further reveal the potential role of hsa_circ_0111277 in the pathology of PE, hsa_circ_0111277 expression in PE placentas (*n* = 25) and normal pregnancy placentas (*n* = 25) was monitored by quantitative real-time PCR. The results revealed that the circ_0111277 expression was clearly enhanced in PE placentas compared with that in normal pregnancy placentas (Fig. 1f). In addition, to provide more supporting evidence, Pearson’s analysis was performed to assess the relationships between hsa_circ_0111277 expression and PE-related factors (proteinuria level at 24 h and placental weight) based on the clinical data of 25 PE patients. As demonstrated in Fig. 1g, h), there were approximately positive correlations of hsa_circ_0111277 expression with both proteinuria level at 24 h (g) and placental weight (h). This indicates that hsa_circ_0111277 may be highly involved in the progression of PE.

### Knockdown of circ_0111277 promoted trophoblast cell migration and invasion

**Fig. 2:**
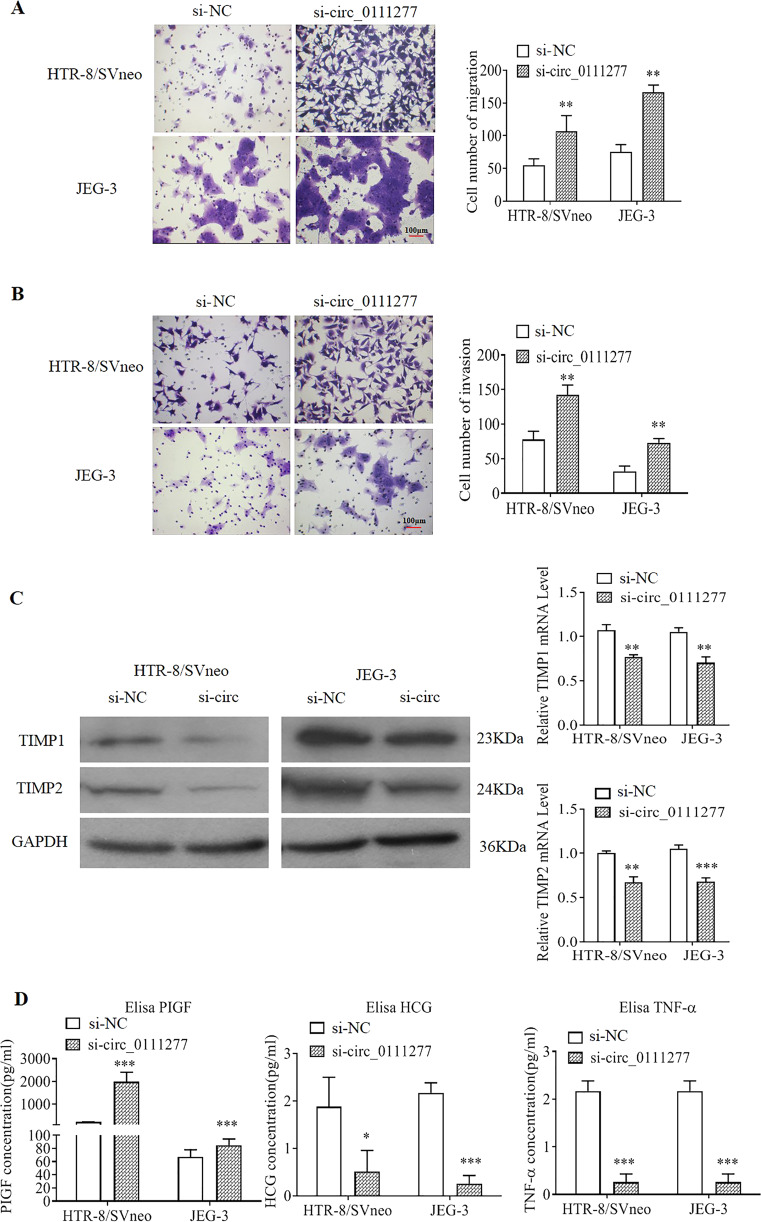
Knockdown of circ_0111277 promotes trophoblast cell migration and invasion. Migration (**a**) and invasion (**b**) capacities of HTR-8/SVneo or JEG-3 cells with or without transient transfection of si-circ_0111277. Nonsense siRNA served as a negative control (si-NC). **c** TIMP1/2 protein levels in HTR-8/SVneo and JEG-3 cells transfected with si-circ_0111277 were determined by western blot assays. **d** TIMP1/2 mRNA levels in HTR-8/SVneo and JEG-3 cells transfected with si-circ_0111277 were measured by qPCR analysis. **e** The concentrations of PLGF, β-hCG, and TNF-α in the supernatants of HTR8/SVneo and JEG-3 cells treated with si-circ_0111277, as determined by ELISA.

Based on the aforementioned results, we concluded that hsa_circ_0111277 may play a pivotal role in PE pathogenesis. Nevertheless, the detailed mechanism concerning its function in PE onset and development was still unclear. It was recognized that placentation failure may mainly be derived from inadequate trophoblast invasion, leading to the occurrence of PE. Hence, to determine whether circ_0111277 participates in PE by modulating trophoblastic invasion and migration, HTR-8/SVneo and JEG-3 cells, as models of Matrigel cell invasion and migration, were both transfected with si-circ_0111277. The results of Transwell assays showed that the downregulation of circ_0111277 markedly promoted the invasion and migration of HTR-8/SVneo and JEG-3 cells when compared with those transfected with empty vectors (Fig. 2a, b). Previous research also indicated that TIMP1/2 secreted by villous cytotrophoblasts, as specific inhibitors of MMP2/9, could contribute to modulating the balance and control of EVT invasion into maternal endomyometrium. In line with this, western blot and q-PCR analyzes revealed variations in the protein and mRNA levels of TIMP1/2, respectively, after downregulation of circ_0111277 via transient transfection with si-circ_0111277 (Fig. 2c, d). The results indicated that TIMP1/2 expression in both HTR-8/SVneo and JEG-3 cells was significantly attenuated by downregulating circ_0111277 levels. The concentrations of placental growth factor (PLGF), free beta human chorionic gonadotrophin (β-hCG), and tumor necrosis factor-α (TNF-α), which were screened as diagnostic tools for PE in a clinical setting, were detected in the supernatants of HTR8/SVneo and JEG-3 cells treated with si-circ_0111277 by ELISA. As indicated in Fig. 2e, the concentrations of PLGF in HTR8/SVneo and JEG-3 cells were clearly enhanced after the knockdown of circ_0111277, while the concentrations of β-hCG and TNF-α dramatically declined upon the same treatment. This revealed that the downregulation of circ_0111277 may play a critical role in the pathogenesis of PE.

### Knockdown of circ_0111277 promoted trophoblast cell migration and invasion by sponging hsa-miR-494-3p

**Fig. 3:**
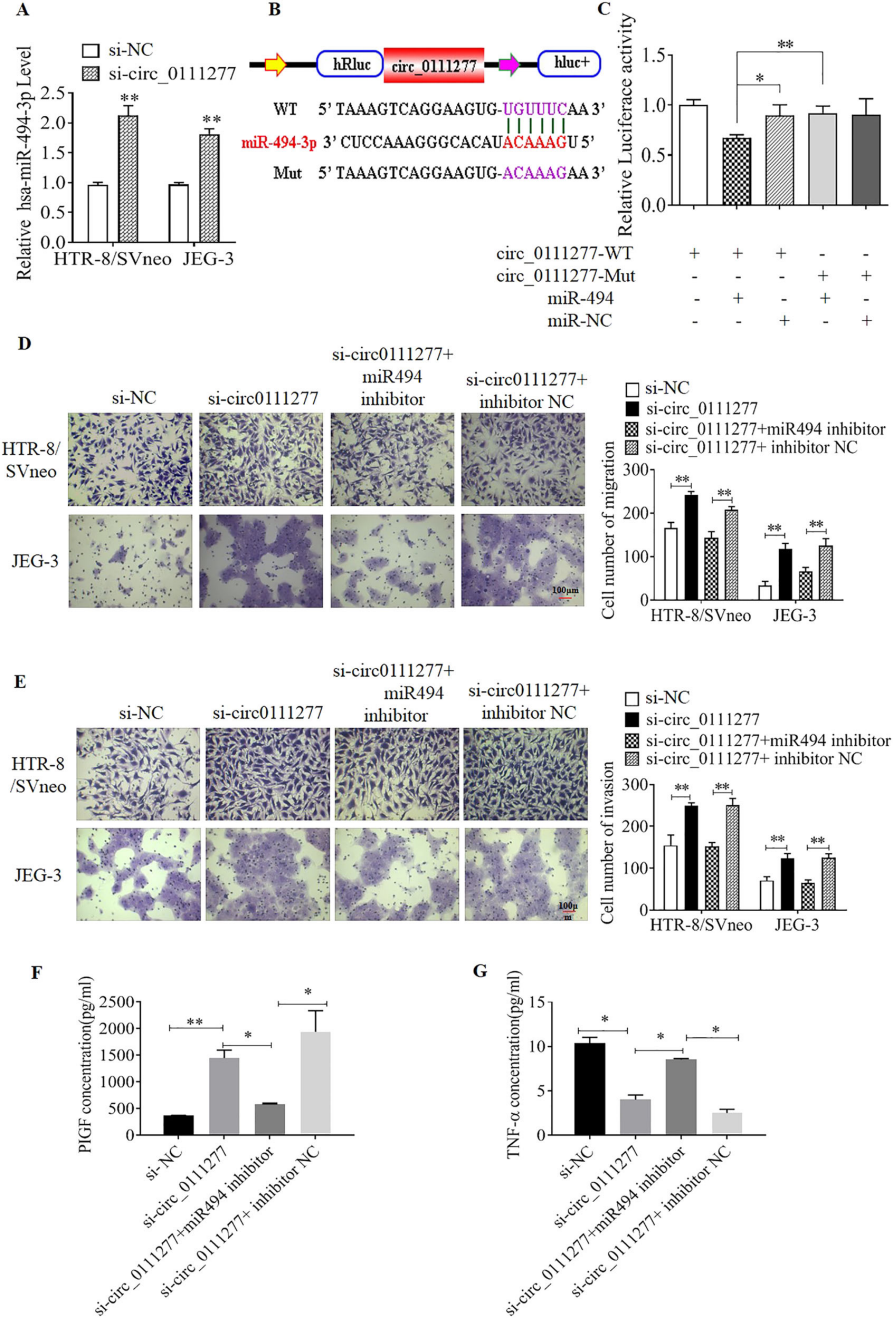
circ_0111277 promotes trophoblast cell migration and invasion by suppressing its sponged target miR-494. Hsa-miR-494-3p level in HTR8/SVneo and JEG-3 cells upon treatment with si-circ_0111277 was examined by qPCR. **a** Schematic illustration of circ_0111277 wild-type (WT) and mutant (Mut) luciferase reporter vectors (pGLO-Firefly-*Renilla* containing cicr0111277 sequence and mutant sequence). The predicted binding sites of miR-494-3p in circ_0111277 are presented in red. **b** miR-494-3p was proven to serve as a sponged target of circ_0111277 by the dual-luciferase reporter assay. **c** Migration **d** and invasion **e** abilities of HTR8/SVneo and JEG-3 cells co-transfected with various vectors as described, including si-circ_0111277, si-circ_0111277+miR-494-3p inhibitor, and si-circ_0111277+miR-494-3p nonsense, were determined by Transwell assays. The concentrations of PLGF (**f**) and TNF-α (**g**) in HTR-8/SVneo cells under the same treatment as described were determined by ELISA.

To determine whether miR-494-3p was the sponged target of circ_0111277, the alterations of hsa-miR-494-3p levels in HTR8/SVneo and JEG-3 cells after downregulation of circ_0111277 were monitored by qPCR assays. As depicted in Fig. 3a, hsa-miR-494-3p levels were remarkably elevated upon using si-circ_0111277 to knockdown circ_0111277 expression, indicating that circ_0111277 can bind to hsa-miR-494-3p. As illustrated in Fig. 3b, the 3′ UTR of *Renilla* luciferase was modified with the wild-type circ_0111277 sequence (WT) or the sequence with mutated binding sites of miR-494 (Mut) to generate luciferase reporter vectors (pGLO-Firefly-*Renilla* containing cicr0111277 sequence and mutant sequence); this further confirmed the interaction between circ_0111277 and miR-494. The luciferase reporter assays showed that there was a clear decline of luciferase activities of WT reporter in 293T cells after the upregulation of miR-494, accompanied by no visible alteration in mutant reporter. All of the above results proved that circ_0111277 could specifically sponge hsa-miR-494 (Fig. 3c). To provide more evidence for the function of miR-494-3p as a sponged target of circ_0111277, Transwell assays were performed to determine whether miR-494-3p overexpression derived from si-circ_0111277 could be abolished by coincubation with miR-494 inhibitor. The results of Transwell assays showed that the migration and invasion of HTR-8/SVneo cells were significantly strengthened upon the employment of si-circ_0111277, whereas such promotion was clearly abolished upon combination with miR-494 inhibitor (Fig. 3d, e). Consistent with this, the concentrations of PLGF and TNF-α in HTR-8/SVneo cells under the aforementioned treatment were determined by ELISA and shown to be consistent with the above results (Fig. 3f, g).

### miR-494 promotes trophoblast cell migration and invasion through targeting HTRA1

**Fig. 4:**
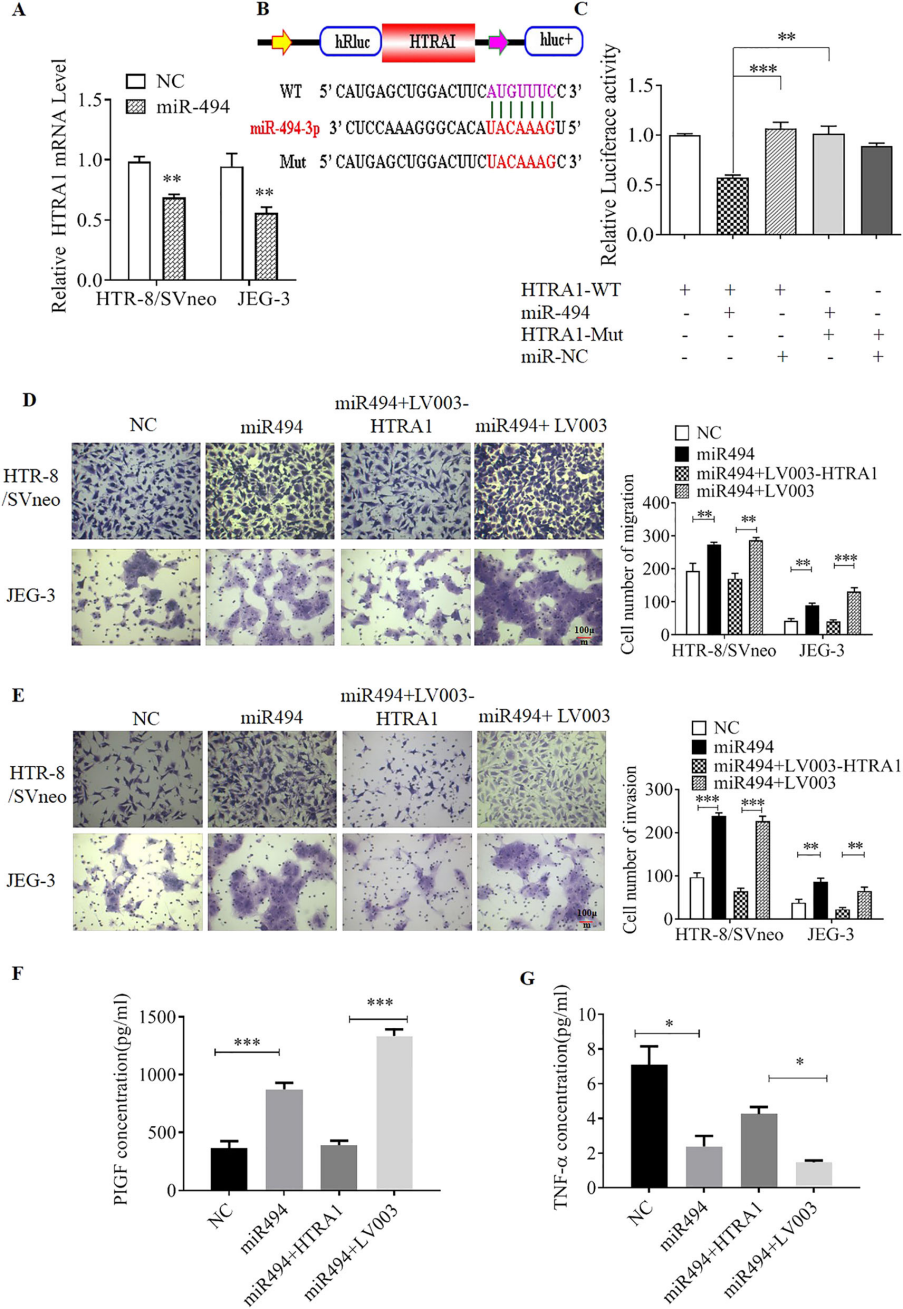
miR-494 facilitates trophoblast cell migration and invasion by targeting HTRA1. HTRA1 mRNA level in HTR8/SVneo and JEG-3 cells after co-transfection with miR-494 mimics was examined by qPCR. **a** Schematic illustration of HTRA1 wild-type (WT) and mutant (Mut) luciferase reporter vector (pGLO-Firefly-*Renilla* containing HTRA1 gene 3′-UTR sequence and mutant sequence). The predicted binding sites of miR-494-3p in circ_0111277 are presented in red. **b** HTRA1 was identified in the downstream pathway of miR-494-3p by the dual-luciferase reporter assay. **c** Migration **d** and invasion **e** abilities of HTR8/SVneo and JEG-3 cells co-transfected with various vectors (NC, miR-494 mimics, miR-494 mimics+pLV3-HTRA1, and miR-494 mimics+pLV3-Ctrl) were determined by Transwell assays. The concentrations of PLGF (**f**) and TNF-α (**g**) in HTR-8/SVneo cells under the same treatment as described were determined by ELISA.

High-temperature requirement-A serine peptidase 1 (HTRA1), a well-known PE marker, is a serine protease that is prominently expressed in the vasculature^21,22^. The sensor domain of loop 3 (L3) and the activation domain of loop D (LD) are essential and highly involved in the trimer-mediated activation of the adjacent HTRA1 subunit. Moreover, the HTRA1 gene has been crucial for unveiling the pathogenesis of diseases of the microvasculature and macrovasculature, especially for PE^23^. HTRA1 was predicted to be a functional component in the downstream pathway of miR-494 based on the TargetScan prediction tool (Supplementary Table 1). Here, to identify whether hsa_circ_0111277 modulates trophoblast cell migration and invasion through the miR-494/HTRA1 pathway, qPCR analysis was performed to examine the alterations of HTRA1 mRNA levels in HTR8/SVneo and JEG-3 cells after co-transfection with miR-494 mimics. As hypothesized, qPCR results revealed that the HTRA1 mRNA level was clearly reduced after the upregulation of miR-494 (Fig. 4a). Here, to identify the interaction between miR-494-3p and HTRA1, the 3′ UTR of *Renilla* luciferase was modified with the wild-type HTRA1 sequence (WT) or the sequence with mutated binding sites of miR-494 (Mut) to construct luciferase reporter vectors (pGLO-Firefly-*Renilla* containing HTRA1 gene 3′-UTR sequence and mutant sequence) as exhibited. As expected, the corresponding luciferase reporter assays revealed that the luciferase activities of a luciferase reporter harboring HTRA1 WT in 293T cells could be clearly downregulated by co-transfection with miR-494 mimics relative to HTRA1 WT, whereas such downregulation failed to be achieved by co-transfection with miR-494 mimics in a luciferase reporter harboring HTRA1 mutation (Fig. 4c). Transwell assays were also conducted to determine the variation of migration and invasion abilities of HTR-8/SVneo and JEG3 cells co-transfected with various vectors as depicted (si-NC, miR-494 mimics, miR-494 mimics+pLV3-HTRA1, and miR-494 mimics+pLV3-Ctrl). As indicated in Fig. 4d, e, we found that cell migration and invasion of 293T cells were clearly promoted by the overexpression of miR-494 mimics when compared with the control vector. Similar promotion was also found in 293T cells co-transfected with miR-494 mimics and pLV3-Ctrl, which served as a control vector of HTRA1-overexpressing plasmid (pLV3-HTRA1). Besides, the Transwell assays demonstrated that the boosted cell migration and invasion in 293T cells triggering the upregulation of miR-494 were remarkably counteracted by the administration of pLV3-HTRA1, validating the identification of HTRA1 function in the downstream pathway of miR-494. Corresponding with this, the concentrations of PLGF and TNF-α in HTR-8/SVneo cells under the same treatment were determined by ELISA. The results showed that PLGF concentrations were drastically enhanced in both HTR-8/SVneo cells co-transfected with either miR-494 mimics or miR-494 mimics and pLV3-Ctrl compared with the level in the control group, while there was no visible alteration in HTR-8/SVneo cells administered miR-494 mimics and pLV3-HTRA1 (Fig. 4f). In contrast, the opposite tendency was observed for the changes of TNF-α concentration under the same treatment conditions (Fig. 4g).

### hsa_circ_0111277 regulates the Notch signaling pathway through the miR-494-3p/HTAR1 axis

**Fig. 5:**
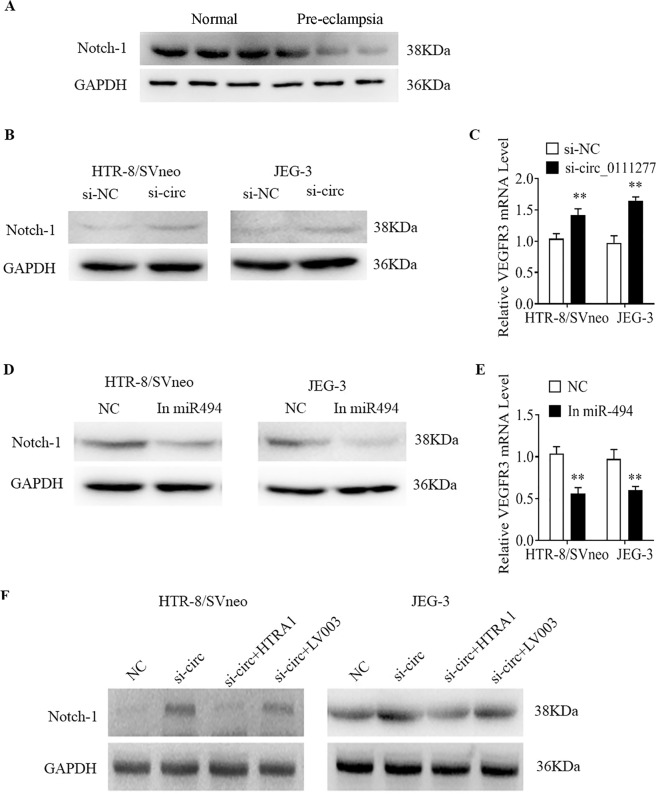
hsa_circ_0111277 regulates the Notch signal pathway through the miR-494-3p/HTAR1 axis. The Notch-1 protein levels between PE placentas (*n* = 3) and normal pregnancy placentas (*n* = 3) were mirrored, as revealed by western blot assays. **a** Notch-1 protein levels in HTR8/SVneo and JEG-3 cells after si-circ_0111277-modulated silencing of hsa_circ_0111277 were measured by western blot assays. **b** VEGFR3 mRNA levels in HTR8/SVneo and JEG-3 cells transfected with si-circ_0111277 were monitored by qPCR. **c** Notch-1 protein **d** and VEGFR3 mRNA levels **e** in HTR8/SVneo and JEG-3 cells upon miR-494 inhibition treatment were measured by western blot and qPCR assays, respectively. **f** Notch-1 protein in HTR8/SVneo and JEG-3 cells co-transfected with si-circ_0111277 or si-circ_0111277+pLV3-HTRA1 was detected by western blot assay.

The Notch signaling pathway is acknowledged to govern differentiation and progression during cell–cell contact in various tissues due to its pivotal function in vascular patterning. Research has shown that Notch signaling plays a critical role in the vascular invasion or migration of trophoblasts. It has been recognized that there is a close interaction between HTAR1 and Notch signaling pathways. Mechanistically, endothelial HTRA1 could modulate the Notch signaling pathway by interacting with the weak Notch ligand JAG1. In addition, VEGFR3 mediated by the Notch pathway, as the main receptor for VEGF-C, is highly involved in embryonic angiogenesis. It has been pointed out by Kitajewski et al. that VEGFR-3 could be upregulated through the Notch pathway in primary human endothelial cells^24^. Hence, to obtain a better understanding of the miR-494-3p/HTAR1 axis mediated by hsa_circ_0111277, we monitored the responses of the Notch-1 pathway upon stimulation with the silencing of hsa_circ_0111277 expression, inhibition of miR-494-3p, or HTAR1 overexpression. The differential expression of Notch-1 protein levels between PE placentas (*n* = 3) and normal pregnancy placentas (*n* = 3) was mirrored by the findings of western blot assays. As depicted in Fig. 5a, the Notch-1 protein levels in PE placentas were substantially alleviated, in contrast to the findings in normal pregnancy placentas, which mirrored the opposite expression patterns of hsa_circ_0111277 presented in Fig. 1f. Meanwhile, higher Notch-1 protein levels in HTR8/SVneo and JEG-3 cells were elicited by si-circ_0111277-modulated silencing of hsa_circ_0111277, which was in accordance with the above results (Fig. 5b). Subsequently, the VEGFR3 mRNA levels in HTR8/SVneo and JEG-3 cells upon applying the same approaches were also determined by qPCR analysis. As shown in Fig. 5c, VEGFR3 mRNA levels in both types of cell were enhanced to a greater extent by downregulating hsa_circ_0111277 levels. The Notch-1 protein and VEGFR3 mRNA levels were also measured in HTR8/SVneo and JEG-3 cells upon miR-494 inhibition. Consistently, Notch-1 protein and VEGFR3 mRNA levels were both alleviated under conditions of miR-494 downregulation compared with those in the control group (Fig. 5d, e). To directly identify whether HTAR1 overexpression can abolish the increased Notch-1 expression triggered by the silencing of hsa_circ_0111277, western blot assay was performed to detect Notch-1 protein in HTR8/SVneo and JEG-3 cells co-transfected with si-circ_0111277 or si-circ_0111277+pLV3-HTRA1. HTR8/SVneo and JEG-3 cells co-transfected with si-circ_0111277+pLV3-Ctrl served as control groups. As shown in Fig. 5f, the gray levels of Notch-1 protein in HTR8/SVneo and JEG-3 cells under the treatment with si-circ_0111277 or si-circ_0111277+pLV3-Ctrl were both higher than those of cells without any treatment. As expected, the elevated Notch-1 protein level as mentioned above was conspicuously attenuated by HTAR1 overexpression, which was in line with the aforementioned results.

## Discussion

At present, circRNAs, a novel type of highly stable and abundant endogenous noncoding RNA, are drawing increasing attention^25^. With the rapid development of massively parallel sequencing technology and bioinformatic analysis, a large number of circRNAs have been identified and shown to participate in the development and progression of various diseases^26,27^. Research has also confirmed that competing endogenous RNAs (ceRNAs) play pivotal roles in the molecular pathology of PE^15,16^. In addition, ceRNA profiling in PE placentas facilitated the identification of more biological processes or pathways deregulated in this condition, which is extremely conducive to the exploration of PE pathogenesis^1^. In this study, we screened out an upregulated circRNA named circ0111277 in placenta, in view of the critical function of PAPPA2 in the pathogenesis of PE. As demonstrated in the above results, circ_0111277 expression was evidently enhanced in PE placentas compared with that in normal pregnancy placentas, which was consistent with upregulated PAPPA2 levels in placentas derived from PE patients. Approximately, positive correlations of hsa_circ_0111277 level with PE-related factors as identified by Pearson’s analysis on 25 PE patients further confirmed this notion.

It has been reported by Dimitriadis et al. that interleukin-11 (IL-11) administered to mice could compromise trophoblast invasion and migration, leading to the development of PE^17^. However, this effect could be efficiently alleviated by silencing PAPPA2 expression. In this study, the invasion and migration of HTR8/SVneo and JEG-3 cells co-transfected with si-circ0111277 were determined by Transwell assays to determine whether the knockdown of circ0111277 could also promote trophoblast invasion and migration. As expected, the depletion of circ0111277 prominently boosted trophoblast invasion and migration. In addition, the regulatory network of circ0111277 with its target genes involved in the initiation and progression of PE needs further exploration. Under the ceRNA hypothesis, it has been asserted that circRNAs serving as ceRNAs could effectively modulate the expression of their miRNA target genes^28^. For example, Xin et al. found that circTNRC18 can sponge miR-762 in trophoblast cells to relieve miR-762 repression of the level of the transcription factor Grhl2, ultimately leading to the development of PE^29^. In our study, we identified that circ0111277 sponged miR-494-3p to alleviate miR-494-3p repression of the level of the transcription factor HTRA1, thus impeding trophoblast invasion and migration.

The HTRA family of serine proteases is crucial for revealing the details of the pathogenesis of various diseases, especially PE. Several studies have revealed that HtrA1 and HtrA3 were both aberrantly expressed in the serum or placental tissues of PE patients, based on analyses of clinical data^30–32^. Based on the TargetScan prediction tool, the screened out gene HTRA1 may serve as a downstream target of miR-494-3p. It was also reported by Oka et al. that insufficient levels of HtrA1 may lead to aberrant placentation and intrauterine growth retardation, thus hindering trophoblast differentiation and preventing remodeling of the maternal arteries^33^. Moreover, Chen et al. revealed that hsa-miR-494-3p can modulate the PI3K/Akt signaling pathway by targeting PTEN^34^. Furthermore, Chen et al.^35^ reported that HtrA4, as a secreted serine protease, was preferentially expressed in trophoblasts, while both HtrA1 and HtrA3 were determined to impede trophoblast invasion, which can counteract the mediation of such invasion by HtrA4. In this study, Transwell assays demonstrated that the promotion of trophoblast invasion and migration resulting from the administration of hsa-miR-494-3p mimics could be efficiently counteracted by upregulating HTRA1, indicating that the hsa_circ_0111277/miR-494-3p/HTRA1 axis plays a vital role in the manipulation of trophoblast invasion. However, the precise mechanism concerning how hsa_circ_0111277 inhibits trophoblast invasion and migration by regulating the miR-494-3p/HTRA1 axis is not yet completely understood. As elaborated by Fischer et al., HTRA1 can effectively regulate Notch signaling by manipulating the level of the antagonistic JAG1 ligand in endothelial cells^36^. In this study, the Notch-1 signal pathway responded to various stimulations, including knockdown of hsa_circ_0111277, inhibition of miR-494-3p, along with the combination of hsa_circ_0111277 silencing and HTRA1 overexpression, which further proved that hsa_circ_0111277 can inhibit trophoblast invasion and migration by modulating the miR-494-3p/HTRA1/Notch-1 axis.

In summary, the data obtained in this study confirmed that the hsa_circ_0111277 level was upregulated in PE placentas compared with that in normal pregnancy placentas, and was highly correlated with PE-related factors. Mechanistically, circ_0111277 sponged hsa-miR-494-3p in trophoblast cells to retard their migration and invasion by regulating the HTRA1/Notch-1 signal pathway. Taking the obtained findings together, the defective trophoblast cell migration and invasion may have been rescued by downregulating the hsa_circ_0111277 level via the miR-494-3p/HTRA1/Notch-1 signal pathway, thereby providing a novel therapeutic target for PE.

## Materials and methods

### Patients and specimens

A cohort of 25 PE patients (*n* = 25) and 25 subjects with normal pregnancies (*n* = 25) admitted to Sun Yat-Sen Memorial Hospital were separately enrolled in this research. Placental tissues were collected promptly from pregnancies undergoing elective cesarean delivery in the absence of labor and preserved in a freezer at −80 °C for later use. According to the American College of Obstetricians and Gynecologists (ACOG), PE is defined as systolic blood pressure of ≥140 mmHg and diastolic blood pressure of ≥90 mmHg with proteinuria of ≥300 mg/day (or a protein/creatinine ratio of ≥0.3 mg/dl or proteinuria of ≥1+) or without proteinuria but with severe clinical features after 20 weeks of gestation in a woman with previously normal blood pressure^37^. Patients complicated with diseases such as diabetes, lupus, urinary tract infection, or chronic renal disease were excluded from this study. The subjects with normal pregnancy were matched to the PE patients in terms of their age and gestational age. This study was approved by the Ethics Committee of Sun Yat-Sen Memorial Hospital and the enrolled subjects provided informed consent to participate in this research. The clinical characteristics of the pregnancies included in this study are presented in Table 1.

**Table 1:** Clinical characteristics of pregnancies recruited in this research.

characteristics	Preeclampsia	Normal control	*P* value
Age (years)	30.87 ± 5.49	31.72 ± 5.3	0.562
BMI	28.46 ± 5.05	25.28 ± 3.04	0.049
Systolic pressure (mmHg)	144 ± 18.12	114.03 ± 8.92	<0.0001
Diastolic pressure (mmHg)	85.93 ± 12.95	74.8 ± 8.04	<0.0001
Platelets	194.39 ± 63.42	221.47 ± 58.85	0.089
Proteinuria level (mg/24)	617 (216, 2747)	0 (0, 0)	<0.0001
Gestational week at delivery	36.42 ± 2.17	39.09 ± 0.55	<0.001
Gestational weeks for confirmed PE	33.52 ± 1.86	–	–
Neonatal Apgar score	8.88 ± 0.65	10	<0.0001
Neonatal weight (kg)	2.74 ± 0.83	3.25 ± 0.29	0.0021

### Cell lines and cell culture

Both 293T cells and human trophoblast cell lines (HTR-8/SVneo and JEG-3 cells) were obtained from the American Type Culture Collection (Manassas, VA, USA). All cell lines were incubated in DMEM consisting of 10% fetal bovine serum and 1% penicillin/streptomycin (Gibco, Shanghai, China) at 37 °C in an incubator with 5% CO_2_.

### Oligonucleotides and overexpression plasmids for transfection

Specific oligonucleotides and plasmids were designed to modulate the expression of circ0111277, miR-494-3p, and HTRA1. Specific siRNAs targeting circ0111277 were obtained from GenePharma (Shanghai, China): si-circ0111277-1, 5′-AAGTCAGGAAGTGTGTTTCAA-3′; si-circ0111277-2, 5′-GTCAGGAAGTGTGTTTCAATA-3′. The full-length circ0111277 and HTRA1 genes were cloned into pLX2 and pVL3 overexpression vectors, respectively (General Biosystems, Anhui, China). In addition, the mimics, inhibitor, and negative controls for hsa-miR-494-3p were obtained from GenePharma (Shanghai, China). The above oligonucleotides and plasmids were all co-transfected into cell lines using Lipofectamine® 2000 Transfection Reagent (#11668019; Life Technologies) based on the manufacturer’s protocol.

### Fluorescence in situ hybridization (FISH)

FISH was carried out to identify the presence of circ_0111277 and its subcellular location by utilizing Cy5-labeled hsa_circ_0111277 probes (5′-CTGGTCTATTGAAACACACTTCCTGACTTTACCCTCTGCA-3′). Briefly, JEG3 cells were first washed with a solution of 0.5% Triton X-100 in 1× PBS, followed by addition of the required volume of a hybridization solution containing Cy5-labeled hsa_circ_0111277 probes after the configuration of probe mixture. Then, hybridization was carried out overnight by coincubation in a humid chamber at 37 °C. The cells were washed with 4× sodium citrate buffer (SSC), 2× SSC, and 1× SSC containing 0.1% Tween-20 three times after removal of the probe mixture, for 5 min each. Subsequently, after washing with 1× PBS for 5 min, the residue was stained with 4′,6-diamidino-2-phenylindole (DAPI) for 20 min in the dark at room temperature. The images were captured by confocal laser scanning microscopy (LSM 800 with Airyscan; Zeiss, Germany). The experiments were repeated three times.

### Cell migration and invasion assays

The cell migration and invasion abilities of HTR8/SVneo and JEG-3 cells under various treatments were determined by Transwell assays by adopting 8 μm pore Transwell chambers without (for migration assays) or with Matrigel (for invasion assays). Briefly, 200 μL of serum-free RPMI 1640 medium was inoculated into the upper chamber at a cell density of 1 × 10^6^/ml, while the lower chamber was filled with complete medium. After 48 h of incubation, the cells in the lower chamber were fixed in 4% paraformaldehyde and stained with crystal violet for 10 min after eradication of the cells in the upper chamber with a cotton swab. Images were obtained under a microscope, with each experiment carried out in triplicate.

### Luciferase reporter assay

For luciferase activity assays, 293T cells were seeded in 24-well plates at a density of 5 × 10^5^ cells per well. Then, they were co-transfected with the depicted plasmids and microRNA mimics or inhibitors using Lipofectamine® 2000 Transfection Reagent for 48 h, in accordance with the manufacturer’s instructions (Life Technologies). Here, the corresponding luciferase reporter plasmids (pGLO-Firefly-*Renilla* containing circ0111277 sequence and mutant sequence, pGLO-Firefly-*Renilla* containing HTRA1 gene 3′-UTR sequence and mutant sequence) were purchased from GenePharma Co. (Shanghai, China). The firefly and *Renilla* luciferase activities were monitored using the Dual-Luciferase Reporter Assay System (Promega, Madison, MA, USA), followed by calculation of the ratio of luminescence from firefly relative to *Renilla* luciferase, which was quantified using a BioTek Synergy HTX multi-mode reader. Each assay was independently conducted in triplicate.

### Concentrations of PLGF, β-hCG, and TNF-α in supernatants determined by ELISA assays

The concentrations of PLGF, β-hCG, and TNF-α in the supernatants of HTR8/SVneo and JEG-3 cells under different treatments were determined by ELISA assays (Cusabio Biotech Co., Ltd., Wuhan, China), in accordance with the manufacturer’s instructions. Briefly, the obtained supernatant samples (50 μL) under various treatments were added to each well of 96-well plates and incubated with the corresponding antibodies for 30 min at 37 °C after fourfold dilution. Subsequently, after washing with PBS as required, 100 μL of TMB solution was added to each well and incubated at 37 °C, followed by addition of the same volume of HRP enzyme-catalyzed solution. After mixing slightly, the obtained solution was incubated in the dark for 15 min at 37 °C prior to the addition of 100 µL of stopping solution per well. Next, the absorbance values were determined using a spectrophotometer (Synergy H4 BioTek, VT) at a wavelength of 450 nm. The concentrations of PLGF, β-hCG, and TNF-α in the supernatants of HTR8/SVneo and JEG-3 cells were measured in accordance with their corresponding standard curves. Experiments were independently performed in triplicate.

### Western blotting analysis

The cells under the described treatments were lysed in RAPI (protein lysis buffer containing protease inhibitor and phosphatase inhibitor) for 30 min on ice. The obtained proteins in the supernatants were then quantified using a BCA kit (Tiangen, Beijing, China). Equal amounts of protein sample (50 µg) were separated through sodium dodecyl sulfate polyacrylamide gel electrophoresis, thus electrically transferring the proteins onto polyvinylidene fluoride membranes (Millipore, Boston, MA). Then, the resultant membranes were blocked with 5% skim milk in TBST buffer for 1 h at room temperature prior to coincubation with the corresponding primary antibodies against TIMP1 and TIMP2 (Proteintech, Wuhan, China) or Notch-1 (Santa Cruz Biotechnology) overnight at 4 °C. Subsequently, the membranes were incubated with secondary antibody (Jackson, West Grove, USA) for 1 h at room temperature. The gray levels of proteins were visualized with horseradish peroxidase-conjugated secondary antibodies via exposure to a chemiluminescence (ECL) detection kit (Solarbio, Beijing, China). The images were obtained using Quality One Imaging software (Bio-Rad, USA). The monoclonal GAPDH antibody (Proteintech, Wuhan, China) served as an internal control.

### qPCR detection

Total RNA from placental tissues and cells was extracted using TRIzol reagent (Invitrogen), followed by the synthesis of cDNA using a reverse transcription kit (Takara, Dalian, China). Power SYBR Green (Takara) in a reaction mix for real-time PCR was utilized to achieve the amplification of cDNA in the ABI7900 system (Applied Biosystems, Waltham, MA, USA). Each reaction was performed in triplicate. The gene expression levels were calculated by normalizing threshold values (Ct) for each mRNA to that of GAPDH mRNA. The specific primers for the real-time PCR were as follows: hsa_circ_0111277, convergent primers (forward, 5′-CCTTTTCCACCATACCACC-3′ and reverse, 5′-ATTCTGCCCCTCGTCCTCT-3′) and divergent primers (forward, 5′-AGTCGATGGATTCCTCTCAT-3′ and reverse, 5′-AAGTTGTTCATCACTCTGTT-3′); hsa-miR-494, forward, 5′-GCCGAGGATACTCGAAGGAGAG-3′ and reverse, 5′-CTCAACTGGTGTCGTGGAGTCGGCAATTCAGTTGAGGATACTAA-3′; HTRA1, forward, 5′-AACTTTATCGCGGACGTGGT-3′ and reverse, 5′-CCGGCACCTCTCGTTTAGAA-3′; TIMP1, forward, 5′-AGTTTTGTGGCTCCCTGGAA-3′ and reverse, 5′-TCCGTCCACAAGCAATGAGT-3′; and TIMP2, forward, 5′-GGCGTTTTGCAATGCAGATG-3′ and reverse, 5′-GAGGGGGCCGTGTAGATAAA-3′.

### Statistical analysis

SPSS software version 20.0 (SPSS Inc., Chicago, IL, USA) or GraphPad Prism 6 was utilized for the statistical analysis of our cohorts. All data are presented as mean ± SD. The significance of differences in hsa_circ_0111277 expression between PE subjects and those with normal pregnancy was identified using t-test. Data of proteinuria level were tested by Mann–Whitney *U* test. In addition, the correlations between hsa_circ_0111277 expression and PE-related factors were assessed using Pearson’s correlation test. *P* values of <0.05 were regarded as statistically significant.

### Ethical statement

All procedures performed in studies involving human participants were in accordance with the ethical standards of the Ethics Committee of Sun Yat-sen Memorial Hospital and with the 1964 Helsinki declaration and its later amendments or comparable ethical standards.

### Informed consent

Informed consent was obtained from all individual participants included in the study.

## Supplementary information

Supplementary table 1
